# β-Asarone Attenuates Aβ-Induced Neuronal Damage in PC12 Cells Overexpressing APPswe by Restoring Autophagic Flux

**DOI:** 10.3389/fphar.2021.701635

**Published:** 2021-07-28

**Authors:** Zhenwan Li, Jin Ma, Zhongsheng Kuang, Yong Jiang

**Affiliations:** The First Clinical Medical College, Guangzhou University of Chinese Medicine, Guangzhou, China

**Keywords:** Alzheimer’s disease, β-asarone, PC12 cells, APPswe, autophagic flux

## Abstract

Alzheimer’s disease (AD) is a neurodegenerative disorder characterized by progressive memory damage and cognitive dysfunction. Studies have shown that defective autophagic flux is associated with neuronal dysfunction. Modulating autophagic activity represents a potential method of combating AD. In Chinese medicine, Acori Tatarinowii Rhizoma is used to treat dementia and amnesia. β-Asarone, an active component of this rhizome can protect PC12 cells from Aβ-induced injury and modulate expression of autophagy factors. However, its cytoprotective mechanisms have yet to be discerned. It is unclear whether β-asarone affects autophagic flux and, if it does, whether this effect can alleviate Aβ cell damage. In the present study, we constructed APPswe-overexpressing PC12 cell line as a cell model of Aβ-induced damage and assessed expression of autophagic flux-related proteins as well as the number and morphology of autophagosomes and autolysosomes. Our results show that β-asarone decreases the expression levels of Beclin-1, p62, LC3-Ⅱ, and Aβ_1-42_. β-Asarone reduced the number of autophagosomes and increased the number of autolysosomes, as determined by confocal laser scanning microscopy and transmission electron microscopy. Our results suggest that β-asarone can protect PC12 cells from Aβ-induced damage by promoting autophagic flux, which may be achieved by enhancing autophagosome-lysosome fusion and/or lysosome function.

## Introduction

Alzheimer’s disease (AD) is a chronic neurodegenerative disease with an insidious onset, and is clinically characterized by generalized dementia, such as memory loss, cognitive dysfunction, and a decline in self-care that ultimately leads to death (Cai et al., 2019). Onset of AD is highly correlated with increasing age. With an aging population and a perpetually increasing life expectancy, the number of patients with AD worldwide has increased each year. In 2018, it was estimated that 50 million people worldwide were living with dementia, with AD accounting for 50–60% of these cases (Potashkin et al., 2019). To date, only five anti-AD drugs have been approved for clinical use by the United States Food and Drug Administration (FDA). These drugs only relieve symptoms but cannot reverse neuronal damage caused by AD (Isla et al., 2021). Therefore, it is critical to understand the pathological mechanisms of AD and to find effective methods of prevention and treatment.

Recently, scientists have realized that the causes of AD are complex and require insight from new scientific perspectives (Palmqvist et al., 2019). Autophagy is a highly conserved intracellular process. It maintains the stability of the intracellular environment by mediating the degradation of abnormal or damaged components through lysosomal degradation (Dasol et al., 2021). Numerous studies have shown that defective autophagy is frequently associated with neuronal dysfunction. Thus, modulation of autophagic activity represents a potential method of preventing and treating AD and other neurodegenerative diseases (Lee et al., 2019).

Autophagy is a dynamic, multi-step process that can be modulated at several steps (Klionsky et al., 2016). The term autophagic flux is used to describe the entire process, including the formation of autophagosomes, the transport of autophagic substrates to lysosomes, and the degradation of autophagic substrates in autolysosomes (Torossian et al., 2019). Measuring the number of autophagosomes at a single time point is insufficient to evaluate the activity of autophagy, results can be misleading because the number of autophagosomes is affected by both formation and clearance (Hu et al., 2017). Therefore, autophagic flux should be measured dynamically and holistically.

When neuronal function is impaired during autophagy, autophagic flux is blocked or autophagosomes accumulate abnormally in the cell. Autophagic dysfunction prevents the timely removal of proteins, such as Aβ, which, in turn, block autophagy. Intracellular accumulation of Aβ affects autophagosome transport, the fusion of autophagosomes and lysosomes, the stability of lysosomal membranes, and the degradation of autophagy substrates, thus promoting the pathological changes seen in AD (Ntsapi et al., 2018; Feng et al., 2020).

Many drugs can regulate the production of autophagosomes. The activation of autophagosomes in the early stage of AD is beneficial as it aids in the clearance of abnormal proteins. However, in lysosomal failure, the fusion of autophagosomes and lysosomes is blocked, and the activation of autophagosomes only increases the accumulation of abnormal proteins. This suggests that drugs that can modulate multiple stages of the autophagic process may be more effective than those that target a single step. Clearing autophagic substrates likely represents a key mechanism necessary to the development of effective therapeutics.

The traditional Chinese medicine Acori Tatarinowii Rhizoma is used widely in the clinic for conditions like dementia and amnesia. β-Asarone, one of its main components, has been shown to pass through the blood-brain barrier (Irie and Keung, 2003). Though studies have illustrated the effects of β-asarone against AD *in vitro* and *in vivo*, its mechanism of action remains elusive (Liang et al., 2015; Saki et al., 2020). In this study, we used PC12 cells, a cell line that was previously derived from a rat pheochromocytoma and has been widely used as an *in vitro* model for neuronal injury (Irie and Keung, 2003; Zhang et al., 2020), to explore whether β-asarone protects from Aβ-induced damage by affecting autophagic flux. Thus, we provide a theoretical basis for the use of β-asarone to improve the symptoms of AD.

## Materials and Methods

### Drug Preparation and Antibodies

β-Asarone was prepared at the Center Lab of the First Affiliated Hospital of Guangzhou University of Chinese Medicine according to the patent “Refined Method of β-asarone” (application number: cn200510100524.9). Its purity was 99.701%, as determined by gas chromatography-mass spectrometry (GC-MS).

Rabbit monoclonal anti-amyloid precursor protein (APP, 76600S), anti-Beclin-1 (3495S), anti-p62 (39749S), anti-Aβ_1-42_ (12843S), anti-β-actin (4970S), and horseradish peroxidase (HRP)-labeled secondary goat anti-rabbit IgG (7074S) antibodies were obtained from Cell Signaling Technology (United States). Rabbit polyclonal anti-LC3 (ab48394) antibody was procured from Abcam (United Kingdom).

### Cell Culture

Rat adrenal pheochromocytoma cells (PC12 cells) were kindly provided by Guangzhou Medical University, China. The cells were cultured in RPMI 1640 media (Gibco, United States) containing 10% (v/v) fetal bovine serum (Gibco, United States) in a humidified atmosphere of 5% CO_2_ at 37°C. 0.25% Trypsin-EDTA (Gibco, United States) was used to passage the cells every 3 days.

### Lentivirus Infection and Stable Selection

PC12 cells stably overexpressing APPswe were constructed by lentivirus infection. Lentiviral particles containing the APPswe gene or an empty vector were designed and packaged by Ubigene Biosciences (Guangzhou, China).

Cells were seeded in 24-well culture plates and, when their density reached 40%, they were cultured in a mixture containing lentiviral particles that was prepared from a lentiviral stock solution containing polybrene (200213A01, Ubigene, China) and complete media. The mixture was replenished 4 h later and replaced with complete media after 24 h. 96 h after infection, 3.5 μg/ml puromycin (1299MG025, Biofroxx, Germany) was added to the media to select stable cell lines. After 72 h, the media was replaced with complete media containing 1.75 μg/ml puromycin, and the cells were expanded for further experimentation. Finally, overexpression of APPswe was confirmed by real-time PCR and Western blot. Aβ_1-42_ was detected by enzyme-linked immunosorbent assay (ELISA).

These stable cell lines were cultured or stored in complete media containing 1.75 μg/ml puromycin to maintain high expression levels of the target gene when they were not in use. During experimentation, they were cultured in complete media to eliminate any possible influence of puromycin.

### Real-Time PCR

The gene expression levels of APP were detected by real-time PCR. Cells were seeded on 6-well culture plates. When the cells reached 40% density, total RNA was extracted using an RNA Isolation kit (R0024, Beyotime, China). Subsequently, total RNA was quantified according to its absorbance, then reverse transcribed into cDNA using a cDNA Synthesis kit (AORT-0020, GeneCopoeia, United States) per manufacturer’s instructions.

Real-time PCR was performed using a qPCR Mix (AOPR-0200, GeneCopoeia) with a CFX96 real-time PCR detection system (Bio-Rad, United States). Primers for APP were obtained from GeneCopoeia (HQP009578). Primers for GAPDH were synthesized by Sangon Biotech (Shanghai, China): 5′-GGG​CTG​CCT​TCT​CTT​GTG​AC-3′ (Forward primer), 5′-CCC​GTT​GAT​GAC​CAG​CTT​CC-3′ (Reverse primer). PCR amplification conditions were: 95°C for 10 min, 40 cycles of denaturation at 95°C for 10 s, annealing at 60°C for 20 s, and extension at 72°C for 15 s. mRNA expression results were analyzed using the 2^−△△CT^ method.

### Western Blot

Protein expression levels of APP, Beclin-1, p62, LC3, Aβ_1-42,_ and β-actin were detected by western blot. After cells were treated for 24 h, they were collected, and total proteins were extracted using RIPA buffer (P0013B, Beyotime) mixed with a protease inhibitor mixture (P1005, Beyotime). Protein concentrations were determined using a BCA assay kit (P0012, Beyotime). Protein samples mixed with loading buffer (P0015, Beyotime) were separated *via* SDS-PAGE (P0012AC, Beyotime) and transferred to PVDF membranes (FFP24, Beyotime). The membranes were blocked with 5% (w/v) BSA (4240GR025, Biofroxx) for 1 h at room temperature and incubated with primary antibodies (1:1,000 dilution) at 4°C overnight. Subsequently, the membranes were treated with HRP-conjugated goat anti-rabbit antibodies (1:1,000 dilution). Finally, the membranes were visualized with an enhanced chemiluminescence reagent kit (WBKLS0100, Millipore, United States) and photographic imaging equipment (ChemiDoc MP, Bio-Rad, United States). Relative band intensities were quantified using Image J software and normalized to β-actin.

## ELISA

The levels of Aβ_1-42_ were measured by ELISA. Based on the results of western blot and real-time PCR, one cell line was selected as the model group. PC12 cells and model cells were passaged and cultured as described above. When cells reached 80% density, the culture media and cells were collected separately. The media was centrifuged at 1,000 × g for 5 min, and the supernatant was collected. Total proteins were extracted by RIPA buffer mixed with protease inhibitor mixture and quantified using a BCA assay kit. The levels of Aβ_1-42_ in culture medium and cell lysate were measured with an ELISA kit (CEA946Hu, Cloud-Clone, Wuhan, China) per manufacturer’s protocol. A microplate reader (MK3, thermal, United States) was used to measure the optical density of the samples at a wavelength of 450 nm. The results were analyzed using Curve expert software, and standard curve regression equations were calculated based on the concentration of the standard density and the optical density values.

### Impedance-Based Cell Analysis

The concentrations and time under which β-asarone and chloroquine diphosphate (CQ, C6628, Sigma, United States) could exert cellular effects were selected by impedance-based cell analysis. Cells were plated onto polylysine-coated (P6407, Sigma) 96-well impedance assay plates (Z96-IMP-96B, Axion, United States). The plate was then placed onto an Axion BioSystems Maestro Z platform (Axion, United States) to monitor impedance in real time. When cells in the normal group reached 40% density (about 40 Ω of impedance), CQ or β-asarone were added at different concentrations, and the impedance was observed and recorded.

### Cell Viability Assay

Cell viability was measured using Cell Counting Kit-8 (CK04, Dojindo, Japan) per manufacturer’s protocol. Briefly, cells were seeded in 96-well culture plates. After cells were treated for 24 h, CCK-8 mixed with complete media at a ratio of 1:9 was added to the plate, which was then incubated for 1 h. Optical density was then measured at 450 nm.

### Confocal Laser Scanning Microscopy Analysis

Cells were seeded in glass-bottom culture dishes (BS-15-GJM, Biosharp, China). When they reached 40% density, mcherry-EGFP-LC3 adenovirus (HB-AP2100001, Hanbio, China) was added per manufacturer’s protocol. After the cells were treated for 24 h, they were fixed with 4% (w/v) paraformaldehyde for 30 min, permeabilized with 0.1% (v/v) tritonX-100 for 15 min, and stained with DAPI (D9542, Sigma) for 10 min in the dark. Finally, fluorescence micrographs were obtained by CLSM (TCS SPE, Leica, Germany). The samples were irradiated by 488, 532, and 405 nm lasers in turn, and the images from all the channels were collected simultaneously. To calculate the number of autophagosomes and autolysosomes, LC3 was tracked by observing the number and distribution of red and green fluorescent dots. For quantification of green and red dots within cells, nine fluorescence micrographs from each group were counted.

### Transmission Electron Microscopy Analysis

Autophagosomes and autolysosomes were imaged using TEM (H-7650, Hitachi, Japan). After cells were treated for 24 h, they were collected and fixed with 3% (w/v) glutaraldehyde, then fixed in 1% (w/v) osmium tetroxide, dehydrated in graded ethanol and acetone solution, and embedded in Epon812 epoxy resin. Sectioning and staining were performed subsequently under general electron microscopy supervision. Finally, ultrathin sections were examined by transmission electron microscopy (TEM). For quantification of autophagosomes and autolysosomes, five electron micrographs from each group were counted.

### Statistical Analysis

All data were analyzed using SPSS 25.0 software. Data that conformed to the normal distribution were expressed as mean ± standard deviation. A Student’s *t*-test was used to analyze the differences between two groups. One-way ANOVA was used for comparison between multiple groups. A Dunnett-t or SNK test was used for pairwise comparison when the variance was homogeneous, and a Dunnett’s T3 test was used when the variance was heterogeneous. *p* < 0.05 was considered statistically significant.

## Results

### Establishment of APPswe-Overexpressing Cell Line

APPswe is an APP mutant with the Swedish mutation that causes increased production of Aβ, resulting in familial AD (Satir et al., 2020). To establish a cell injury model that is induced by Aβ, PC12 cells were infected with lentiviral particles containing APPswe, with an empty vector used to generate a control line (herein termed “virus control group”). We identified four stable cell lines expressing APPswe by screening with puromycin. Gene and protein expression levels of APP were detected by real-time PCR and western blot to confirm that these cell lines stably overexpressed APPswe.

**FIGURE 1:**
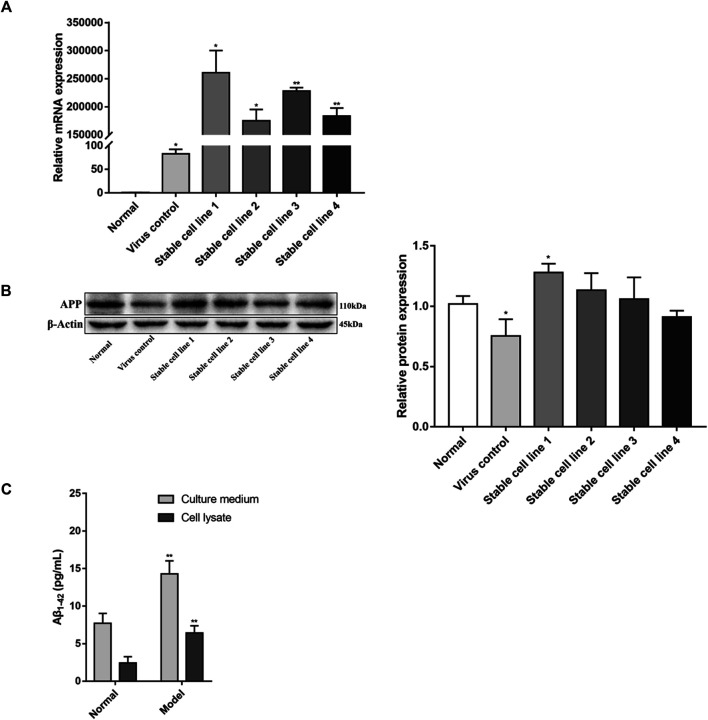
Identification of APPswe-overexpressing cell line. Five stable cell lines were obtained by viral infection and screening, four of which were infected by lentivirus containing the APPswe gene. The other cell line was infected with an empty vector. The expression levels of APP were determined by **(A)** real-time PCR (compared with the normal group, ^*^ *p* < 0.05, ^**^ *p* < 0.01, *n* = 3) and **(B)** western blot (compared with the normal group, ^*^ *p* < 0.05, *n* = 4). **(C)** The levels of Aβ_1-42_ were measured by ELISA (compared with the normal group, ^**^ *p* < 0.01, *n* = 6).

Compared with the normal group, the expression of APP mRNA was significantly increased (*p* < 0.05, *p* < 0.01, Figure 1A) in all four stable cell lines. APP protein expression was also significantly increased (*p* < 0.05, Figure 1B) in stable cell line 1, while there was no difference in the remaining stable cell lines (*p* > 0.05). Based on these results, stable cell line 1 was selected as a model for subsequent experimentation (herein termed “model group”).

Compared with the normal group, the expression of APP mRNA in the virus control group was increased (*p* < 0.05, Figure 1A), but the protein expression of APP in the virus control group was decreased (*p* < 0.05, Figure 1B). With regards to cell viability, there was no significant difference between the virus control group and normal group (*p* > 0.05, Figure 3A). This suggests that lentiviral infection may have an effect on the expression of APP, but this effect is negligible for PC12 cells.

The levels of Aβ_1-42_ in APPswe-overexpressing cell line were detected by ELISA. As shown in Figure 1C, in both culture media and cell lysates, the levels of Aβ_1-42_ in the model group were increased compared with the normal group (*p* < 0.01).

### Determination of β-Asarone and CQ Concentrations and Time of Exposure

**FIGURE 2:**
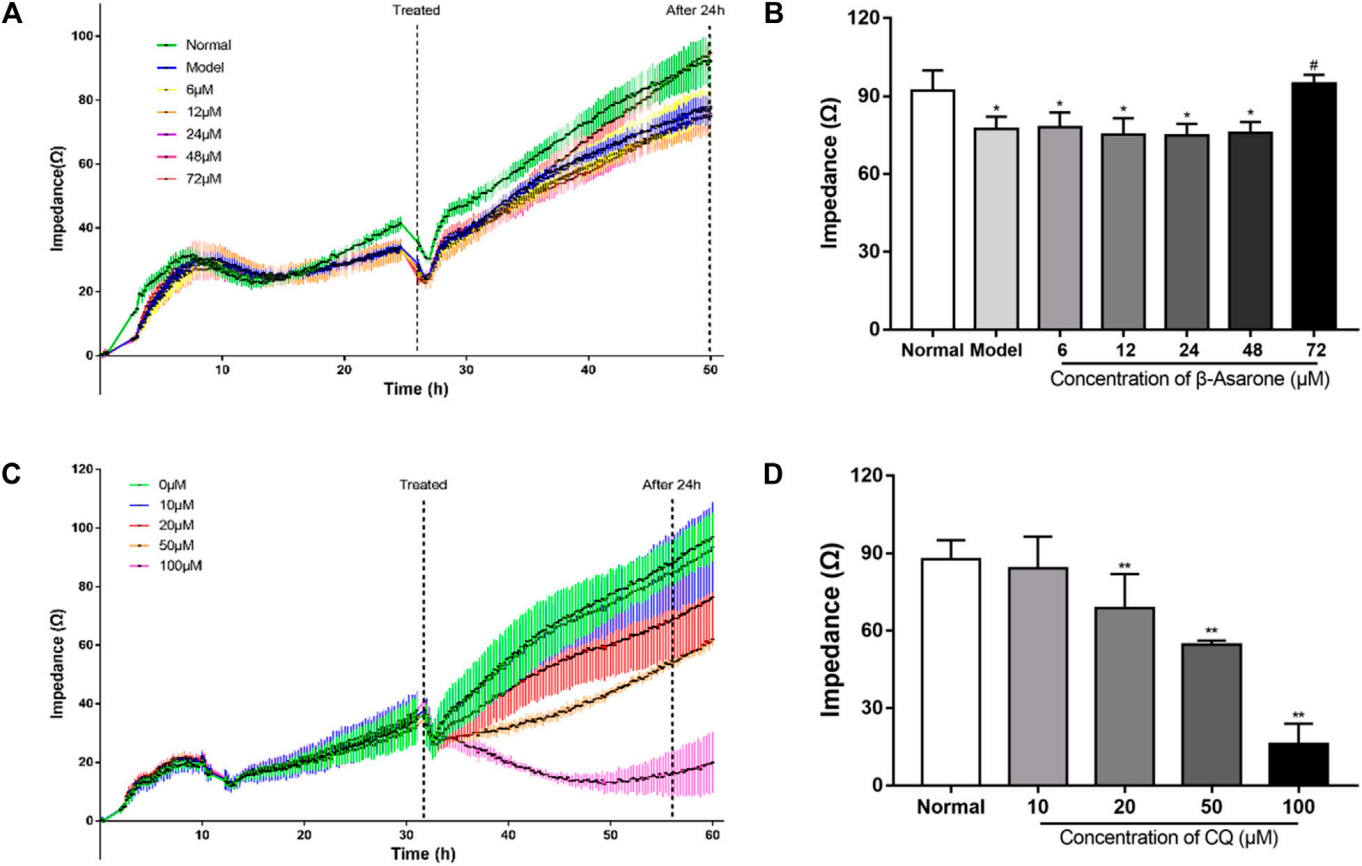
Determination of the optimal administration conditions for drugs. **(A, B)** The concentration and time of β-asarone were determined by impedance-based cell analysis (compared with the normal group, ^*^ *p* < 0.05; compared with the model group, ^#^ *p* < 0.05, *n* = 6). **(C, D)** The concentration and time of CQ were determined by impedance-based cell analysis (compared with the normal group, ^**^ *p* < 0.01, *n* = 6).

Impedance-based cell analysis was used to identify the conditions where β-asarone might exert its physiological effect. As shown in Figure 2A, the impedance of each group was similar within 17 h after cell seeding. But after 17 h, the impedance of the normal group with PC12 cells began to increase further, compared with the other groups with APPswe-overexpressing cells, and the difference continuously expanded with the extension of time. With the addition of β-asarone in APPswe-overexpressing cells, the impedance of the 72 μM group began to rise further compared with the other groups with different concentration gradients, and the difference continuously widened over time. At 24 h after β-asarone administration, there was no difference between the normal group and 72 μM group (*p* > 0.05, Figure 2B). The impedance of the model group and groups treated with 6, 12, 24, 48 μM β-asarone were significantly decreased compared with the normal group (*p* < 0.05). Compared with the model group, the impedance of the 72 μM group was increased (*p* < 0.05). And there was no significant difference in impedance between the model group and groups treated with 6, 12, 24, 48 μM β-asarone (*p* > 0.05). Based on these results, 72 μM β-asarone treatment for 24 h was chosen for subsequent experimentation.

In determining optimal conditions for CQ treatment, we found that impedance decreased in a dose-dependent manner. The difference in impedance among the groups reached its maximum when the drug was added for 24 h (Figure 2D). Compared with the normal group, the impedance of the groups treated with 20, 50, or 100 μM CQ was decreased (*p* < 0.01); however, treatment with 10 μM of CQ had no significant effect (*p* > 0.05). Therefore, 20 μM CQ treatment for 24 h was chosen for subsequent experimentation.

The impedance showed a fast and transient decrease during the assay. That is because the impedance assay plates were temporarily removed from the platform for observation of cell growth or adding drugs. From Figures 2A,C, when the impedance assay plates were put back to platform, the impedance of each group was restored to the level of before being removed in a short time. Therefore, the effects of these operations and the resulting fluctuations on the assay can be considered to be negligible.

### β-Asarone Protects Cells From Aβ

**FIGURE 3:**
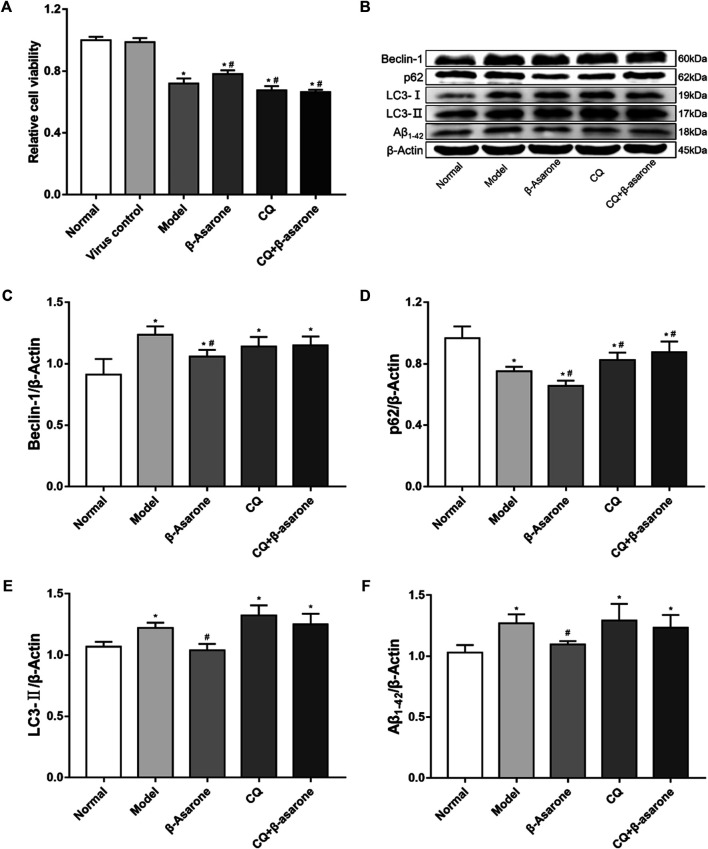
Alterations in cell viability and expression of autophagic flux-related proteins. **(A)** The effect of β-asarone on cell viability was determined by CCK-8 assay (compared with the normal group, ^*^ *p* < 0.05; compared with the model group, ^#^ *p* < 0.05, *n* = 6). **(B–F)** The effect of β-asarone on autophagic flux-related proteins was assessed by western blot (compared with the normal group, ^*^ *p* < 0.05; compared with the model group, ^#^ *p* < 0.05, n ≥ 4).

Our results show that the viability of cells in the model group, β-asarone group, CQ group, and CQ + β-asarone group was decreased (*p* < 0.05). Compared with the model group, the viability of the β-asarone group increased significantly (*p* < 0.05), while in the CQ group and CQ + β-asarone group, viability decreased (*p* < 0.05). There was no difference between the normal group and virus control group (*p* > 0.05) (Figure 3A).

### Changes in Expression of Autophagic Flux-Related Proteins

Beclin-1, p62, LC3, and Aβ_1-42_ were detected from cell lysates by western blot (Figures 3B–F). Our results show that compared with the normal group, the expression levels of Beclin-1, LC3-II, and Aβ_1-42_ were increased (*p* < 0.05) in the model group; however, expression levels of p62 were decreased (*p* < 0.05) in the model group. Compared with the model group, the expression levels of p62 were increased in the CQ group and the CQ + β-asarone group (*p* < 0.05), but there was no statistically significant difference in the levels of either Beclin-1, LC3-Ⅱ or Aβ_1-42_ (*p* > 0.05). Furthermore, the levels of Beclin-1, p62, LC3-Ⅱ and Aβ_1-42_ were decreased in the β-asarone group, compared with the model group (*p* < 0.05). There was no significant difference in the protein expression levels of Beclin-1, p62, LC3-Ⅱ or Aβ_1-42_ between the CQ and CQ + β-asarone groups (*p* > 0.05).

### Analysis of Autophagosomes and Autolysosomes Status

To evaluate autophagic flux, we infected cells with mCherry-EGFP-LC3 adenovirus to visualize autophagosomes. In our assay, the intracellular LC3 simultaneously carried two kinds of fluorescent proteins, mCherry and EGFP. The green fluorescence of EGFP will be quenched in an acidic environment (pH < 5) due to its acid-sensitive nature, so a yellow fluorescent dot (the superposition of red and green fluorescence) represents an autophagosome or an alkalized autolysosome, and a single red fluorescent dot represents a normal autolysosome.

**FIGURE 4:**
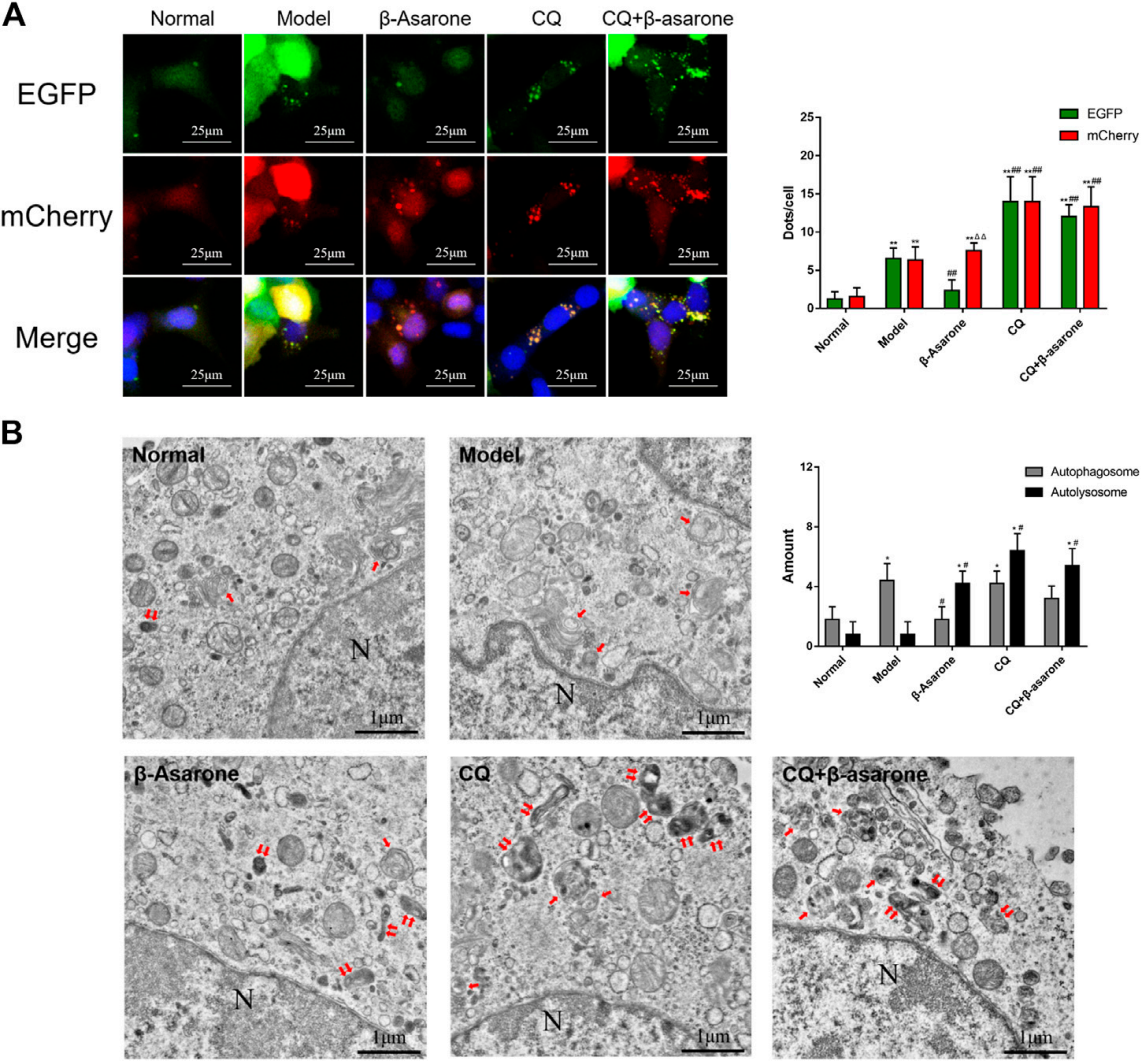
Effects of β-asarone on status of autophagosomes and autolysosomes. **(A)** Cells were infected by an mCherry-EGFP-LC3 adenovirus and fluorescence was observed by CLSM (600×) (compared with the normal group, ^**^ *p* < 0.01; compared with the model group, ^##^ *p* < 0.01; compared with green fluorescent dots, ^ΔΔ^ *P*<0.01, *n* = 9). **(B)** Autophagosomes and autolysosomes were detected by TEM (“↑” points to an autophagosome, “↑↑” points to an autolysosome, “N” point to a nucleus, 20,000×) (compared with the normal group, *p < 0.05; compared with the model group, ^#^ *p* < 0.05, *n* = 5).

In the normal group, few fluorescent dots were observed with two fluorescence channels (Figure 4A). Compared with the normal group, the number of green and red fluorescent dots was significantly increased in the model group (*p* < 0.01). The number of green and red fluorescent dots in the model group was similar (*p* > 0.05). Compared with the model group, the number of red fluorescent dots was unchanged (*p* > 0.05) in the β-asarone group, while the number of green dots decreased significantly (*p* < 0.01). The number of two-color fluorescent dots was further increased in the CQ group, while the number of green and red fluorescent dots was unchanged (*p >* 0.05). Compared with the CQ group, there was no significant difference in two-color dots in the CQ + β-asarone group (*p* > 0.05).

Next, we performed TEM to confirm the number of autophagosomes and autolysosomes (Figure 4B). The number of autophagosomes and autolysosomes in the normal group were relatively low. Compared with the normal group, the number of autophagosomes in the model group increased significantly (*p* < 0.05), while the number of autolysosomes remained unchanged (*p* > 0.05). Compared with the model group, the number of autophagosomes in the β-asarone group decreased (*p* < 0.05), while the number of autolysosomes increased (*p* < 0.05). Compared with the model group, the morphology of autophagosomes and autolysosomes in the CQ group became disordered and enlarged, and their number increased significantly (*p* < 0.05). Compared with the CQ group, there was no change in autophagosome and autolysosome number or morphology in the CQ + β-asarone group (*p* > 0.05).

## Discussion

The etiology and molecular mechanisms of AD are still unclear. The amyloid cascade hypothesis was proposed based on the pathological features of senile plaques (SPs) formed by the deposition of Aβ. This hypothesis suggests that the aggregation of Aβ can damage neurons through multiple pathways and mechanisms, induce apoptosis, and lead to AD (Karran and De Strooper, 2016). Aβ is a small molecular polypeptide composed of 37–43 amino acids that is produced by the hydrolysis of the amyloid precursor protein (APP) by β-secretase and γ-secretase endogenous proteases (Velez et al., 2019). We used APPswe, an APP mutant with a tandem double mutation (K670N/M671L) to enhance the recognition function of β-secretase, which leads to increased Aβ production (Satir et al., 2020).

We constructed a stable APPswe-overexpressing PC12 cell line which showed enhanced expression levels of Aβ_1-42_. Compared with previous models of Aβ dysregulation, our model can generate endogenous Aβ (Wong et al., 2020). Transgenic AD mouse models like this have been used previously (Bolognin et al., 2012; Jura et al., 2019). Thus, our results can be used as a basis for further *in vivo* studies.

CQ is the only FDA-approved drug that inhibits autophagy (Zhang et al., 2020). Its function in blocking autophagic flux is to prevent autophagosomes from fusing with lysosomes to form autolysosomes (Mauthe et al., 2018; Fedele and Proud, 2020). To explore which step of autophagy might be affected by β-asarone, CQ was used. We hypothesized the following: if β-asarone can directly reverse the blockage in autophagic flux caused by CQ, then it can potentially enhance autophagosome-lysosome fusion. In contrast, if β-asarone failed to reverse autophagic flux blockade caused by CQ, then autophagosomes would be unable to fuse with lysosome and would then be degraded. Our results show that when autophagic flux was blocked by CQ, the number of autophagosomes did not significantly change, suggesting that β-asarone has no obvious effect on the formation of autophagosomes. Further comparison between the β-asarone group and model group showed that β-asarone significantly reduced the number of autophagosomes in Aβ-damaged cells. Thus, it could be speculated that β-asarone may promote the degradation of autophagosomes in Aβ-damaged cells.

To evaluate the activity of autophagic flux and explore the mechanism of β-asarone’s protective effects, the expression levels of Beclin-1, p62, LC3-Ⅱ, and Aβ_1-42_ were assessed. Beclin-1 (BECN1) initiates the formation of autophagosomes and induces the recruitment of other autophagy-related proteins to the autophagosome (Wang et al., 2017). Therefore, Beclin-1 protein can serve as a marker for autophagy, and its expression levels can be used to assess whether autophagosome production is activated or inhibited. Microtubule-associated protein 1 light chain 3 (LC3) is highly associated with the formation of the autophagosome (Sun et al., 2017). After autophagosome formation, LC3-Ⅱ remains tightly bound to the autophagosome membrane until the fusion of autophagosome and lysosome. Therefore, the expression levels of LC3-Ⅱ or the ratio of LC3-Ⅱ/LC3-Ⅰ can be used to evaluate the number of intracellular autophagosomes (Wang et al., 2013). Sequestosome 1 (SQSTM1)/p62 (p62) is a selective autophagic substrate, with several functional domains such as ubiquitin-associated (UBA) and LC3-interacting region (LIR). Therefore, p62 can be regarded as a bridge between LC3 and protein ubiquitination, allowing ubiquitinated proteins to be targeted to the autophagosome for degradation (Zhang et al., 2019). Both Aβ_1-42_ and Aβ_1-40_ are derived from APP after cleavage by β-secretase and γ-secretase. Of the two, Aβ_1-42_ is more prone to oligomerization and aggregation and is more neurotoxic (Chun et al., 2020). Therefore, we used Aβ_1-42_ to evaluate the protective effects of β-asarone on Aβ-damaged cells. In addition, colocalization of Aβ with autophagosomes and lysosomes has been previously observed, which suggests that Aβ is a potential autophagic substrate and may be used to evaluate the activity of autophagic flux (Chen et al., 2016; Cheng et al., 2019).

In our study, autophagy was maintained at a low level in normal cells, a finding consistent with previous studies (López-Pérez et al., 2019). In PC12 cells damaged by Aβ due to overexpression of APPswe, increased expression of Beclin-1 may represent the stress response of cells, as they attempt to increase the number of autophagosomes to eliminate excess Aβ_1-42_ (Jiang et al., 2018). High expression levels of LC3-Ⅱ indicates that the number of autophagosomes has increased, which may be caused by the stress-induced response of autophagy, the blocked fusion of autophagosomes and lysosomes, or the inability of autophagosomes to degrade substrates, among other causes (Shen et al., 2020). We found that a large number of autophagosomes or alkalized autolysosomes accumulated APPswe-overexpressing cells, suggesting that autophagic flux was blocked, which might have been caused by the blocked fusion of autophagosomes and lysosomes or disruption of lysosomal function (Chen et al., 2019). Moreover, decreased expression levels of p62 were observed in these cells, a finding that is inconsistent with other studies (Tanji et al., 2014). This may represent increased autophagic flux, or Aβ may have inhibited the production of p62 (Gu et al., 2018).

When APPswe-overexpressing cells were co-cultured with β-asarone, the expression levels of Beclin-1, p62, LC3-Ⅱ, and Aβ_1-42_ decreased. The decrease in Aβ_1-42_ suggests that the protective effect of β-asarone may be due to an enhanced clearance of Aβ_1-42_ (Wang et al., 2019). The decrease in Beclin-1 may be due to the elimination of Aβ_1-42_ from cells due to β-asarone. When the cause of stress state was relieved, the expression of Beclin-1 tended to return to normal level (Deng et al., 2020). The decreased levels of p62 and LC3-Ⅱ suggest that the number of autophagosomes decreased, indicating that β-asarone promoted the clearance of Aβ_1-42_ by promoting autophagosome-lysosome fusion or autolysosomal degradation (Barbero-Camps et al., 2018). The observation that the number of autophagosomes or alkalized autolysosomes decreased, while the number of normal autolysosomes increased, suggests that β-asarone can ameliorate dysregulation of autophagic flux caused by Aβ by promoting autophagosome-lysosome fusion or enhancing lysosomal fusion (Barbero-Camps et al., 2018).

When APPswe-overexpressing cells were co-cultured with CQ, the expression levels of p62 increased, suggesting an increase in the number of autophagosomes and inhibition of autophagic flux (Lin et al., 2019). Additionally, the expression levels of Beclin-1, LC3-Ⅱ, and Aβ_1-42_ did not change, and the accumulation of autophagosomes or alkalized autolysosomes was also increased, confirming this effect (Sun et al., 2020). When APPswe-overexpressing cells were co-cultured with CQ and β-asarone, there was no significant difference in the expression of Beclin-1, p62, LC3-Ⅱ, and Aβ_1-42_. The status of autophagosomes and autolysosomes were no significant change under microscope. These results showed that β-asarone did not improve the inhibitory effect on autophagic flux induced by CQ. Thus, β-asarone did not regulate autophagosome production. We speculate that β-asarone may promote autophagosome degradation instead.

In summary, we found that Aβ inhibits autophagic flux, which may be due to inhibition of autophagosome-lysosome fusion or disruption of lysosomal function (Han et al., 2020). We found that β-asarone may protect against Aβ-induced damage by promoting autophagosome-lysosome fusion and/or lysosomal function, thus enhancing autophagic flux and promoting the elimination of Aβ. As discussed by Sasaguri, each AD model has its comparative strengths and limitations. We choose our model with the scientific and therapeutic goal of a prospective preclinical study (Sasaguri et al., 2017). Limitations of our model include the fact that APP overexpression can result in the overproduction of APP and other proteolytic fragments in addition to Aβ. Other APP metabolites have been shown to affect synaptic transmission and they could potentially interfere with autophagy (Andrade-Talavera and Rodríguez-Moreno, 2021). It is crucial to translate our findings to in vivo-based models and functional molecular studies. In future studies, we will detect the effects of these metabolites on autophagic flux to provide a more robust scientific basis for finding neuroprotective monomers from traditional Chinese medicine.

## Data Availability

The raw data supporting the conclusions of this article will be made available by the authors, without undue reservation.

## References

[B1] Andrade-Talavera Y. Rodríguez-Moreno A. (2021). Synaptic Plasticity and Oscillations in Alzheimer's Disease: A Complex Picture of a Multifaceted Disease. Front. Mol. Neurosci. 14. 10.3389/fnmol.2021.696476 PMC8248350 34220451

[B2] Barbero-Camps E. Roca-Agujetas V. Bartolessis I. de Dios C. Fernández-Checa J. C. Marí M. (2018). Cholesterol Impairs Autophagy-Mediated Clearance of Amyloid Beta while Promoting its Secretion. Autophagy 14 (7), 1129–1154. 10.1080/15548627.2018.1438807 29862881 PMC6103708

[B3] Bolognin S. Blanchard J. Wang X. Basurto-Islas G. Tung Y. C. Kohlbrenner E. (2012). An Experimental Rat Model of Sporadic Alzheimer's Disease and rescue of Cognitive Impairment with a Neurotrophic Peptide. Acta Neuropathol. 123 (1), 133–151. 10.1007/s00401-011-0908-x 22083255 PMC3889170

[B4] Cai M. Lee J.-H. Yang E. J. (2019). Electroacupuncture Attenuates Cognition Impairment via Anti-neuroinflammation in an Alzheimer's Disease Animal Model. J. Neuroinflammation 16 (1), 264. 10.1186/s12974-019-1665-3 31836020 PMC6909515

[B5] Chen F.-J. Liu B. Wu Q. Liu J. Xu Y.-Y. Zhou S.-Y. (2019). Icariin Delays Brain Aging in Senescence-Accelerated Mouse Prone 8 (SAMP8) Model via Inhibiting Autophagy. J. Pharmacol. Exp. Ther. 369 (1), 121–128. 10.1124/jpet.118.253310 30837279

[B6] Chen X. Wagener J. F. Ghribi O. Geiger J. D. (2016). Role of Endolysosomes in Skeletal Muscle Pathology Observed in a Cholesterol-Fed Rabbit Model of Alzheimer's Disease. Front. Aging Neurosci. 8, 129. 10.3389/fnagi.2016.00129 27375475 PMC4896918

[B7] Cheng D. Tan Q. Zhu Q. Zhang J. Han X. Fang P. (2019). TFEB Probably Involved in Midazolam-Disturbed Lysosomal Homeostasis and its Induced β-Amyloid Accumulation. Front. Hum. Neurosci. 13, 108. 10.3389/fnhum.2019.00108 31164812 PMC6536689

[B8] Chun Y. S. Cho Y. Y. Kwon O. H. Zhao D. Yang H. O. Chung S. (2020). Substrate-Specific Activation of α-Secretase by 7-Deoxy-Trans-Dihydronarciclasine Increases Non-amyloidogenic Processing of β-Amyloid Protein Precursor. Molecules 25 (3), 646. 10.3390/molecules25030646 PMC7037359 32028607

[B9] Dasol K. Hui-Yun H. Sun J. E. Young K. J. Shin Y. J. Jeong K. H. (2021). Activation of Mitochondrial TUFM Ameliorates Metabolic Dysregulation through Coordinating Autophagy Induction. Commun. Biol. 4 (1), 1. 10.1038/s42003-020-01566-0 33398033 PMC7782552

[B10] Deng M. Huang L. Zhong X. (2020). Β-asarone Modulates Beclin-1, LC3 and P-62 E-xpression to A-ttenuate Aβ40 and Aβ42 L-evels in APP/PS1 T-ransgenic M-ice with Alzheimer's D-isease. Mol. Med. Rep. 21 (5), 2095–2102. 10.3892/mmr.2020.11026 32186763 PMC7115210

[B11] Fedele A. O. Proud C. G. (2020). Chloroquine and Bafilomycin A Mimic Lysosomal Storage Disorders and Impair mTORC1 Signalling. Biosci. Rep. 40 (4). 10.1042/BSR20200905 PMC7189491 32285908

[B12] Feng Q. Luo Y. Zhang X.-N. Yang X.-F. Hong X.-Y. Sun D.-S. (2020). MAPT/Tau Accumulation Represses Autophagy Flux by Disrupting IST1-Regulated ESCRT-III Complex Formation: a Vicious Cycle in Alzheimer Neurodegeneration. Autophagy 16 (4), 641–658. 10.1080/15548627.2019.1633862 31223056 PMC7138218

[B13] Gu L. Yu Q. Li Q. Zhang L. Lu H. Zhang X. (2018). Andrographolide Protects PC12 Cells against β-Amyloid-Induced Autophagy-Associated Cell Death through Activation of the Nrf2-Mediated P62 Signaling Pathway. Ijms 19 (9), 2844. 10.3390/ijms19092844 PMC6165383 30235892

[B14] Han K. Kim S. H. Choi M. (2020). Computational Modeling of the Effects of Autophagy on Amyloid-β Peptide Levels. Theor. Biol. Med. Model. 17 (1), 2. 10.1186/s12976-020-00119-6 32102666 PMC7045373

[B15] Hu J. Meng Y. Zhang Z. Yan Q. Jiang X. Lv Z. (2017). MARCH5 RNA Promotes Autophagy, Migration, and Invasion of Ovarian Cancer Cells. Autophagy 13 (2), 333–344. 10.1080/15548627.2016.1256520 27875077 PMC5324849

[B16] Irie Y. Keung W. M. (2003). Rhizoma Acori Graminei and its Active Principles Protect PC-12 Cells from the Toxic Effect of Amyloid-β Peptide. Brain Res. 963 (1), 282–289. 10.1016/S0006-8993(02)04050-7 12560134

[B17] Isla A. G. Balleza-Tapia H. Fisahn A. (2021). Efficacy of Preclinical Pharmacological Interventions against Alterations of Neuronal Network Oscillations in Alzheimer's Disease: A Systematic Review. Exp. Neurol. 343, 113743. 10.1016/j.expneurol.2021.113743 34000250

[B18] Jiang S. Zhao Y. Zhang T. Lan J. Yang J. Yuan L. (2018). Galantamine Inhibits β-amyloid-induced Cytostatic Autophagy in PC12 Cells through Decreasing ROS Production. Cell Prolif 51 (3), e12427. 10.1111/cpr.12427 29292543 PMC6528845

[B19] Jura B. Macrez N. Meyrand P. Bem T. (2019). Deficit in Hippocampal Ripples Does Not Preclude Spatial Memory Formation in APP/PS1 Mice. Sci. Rep. 9 (1), 20129. 10.1038/s41598-019-56582-w 31882821 PMC6934724

[B20] Karran E. De Strooper B. (2016). The Amyloid cascade Hypothesis: Are We Poised for success or Failure?. J. Neurochem. 139 (Suppl. 2), 237–252. 10.1111/jnc.13632 27255958

[B21] Klionsky D. J. Abdelmohsen K. Abe A. Abedin M. J. Abeliovich H. Acevedo Arozena A. (2016). Guidelines for the Use and Interpretation of Assays for Monitoring Autophagy (3rd Edition). Autophagy 12 (1), 1–222. 10.1080/15548627.2015.110035610.1080/15548627.2016.1139264 26799652 PMC4835977

[B22] Lee H. J. Lee J. O. Lee Y. W. Kim S. A. Seo I. H. Han J. A. (2019). LIF, a Novel Myokine, Protects against Amyloid-Beta-Induced Neurotoxicity via Akt-Mediated Autophagy Signaling in Hippocampal Cells. Int. J. Neuropsychopharmacol. 22 (6), 402–414. 10.1093/ijnp/pyz016 31125414 PMC6545540

[B23] Liang Z.-h. Cheng X.-h. Ruan Z.-g. Wang H. Li S.-s. Liu J. (2015). Protective Effects of Components of the Chinese Herb Grassleaf Sweetflag Rhizome on PC12 Cells Incubated with Amyloid-Beta42. Neural Regen. Res. 10 (8), 1292–1297. 10.4103/1673-5374.162762 26487858 PMC4590243

[B24] Lin Y. Wu C. Wang X. Kemper T. Squire A. Gunzer M. (2019). Hepatitis B Virus Is Degraded by Autophagosome-Lysosome Fusion Mediated by Rab7 and Related Components. Protein Cell 10 (1), 60–66. 10.1007/s13238-018-0555-2 29876903 PMC6321816

[B25] López-Pérez Ó. Otero A. Filali H. Sanz-Rubio D. Toivonen J. M. Zaragoza P. (2019). Dysregulation of Autophagy in the central Nervous System of Sheep Naturally Infected with Classical Scrapie. Sci. Rep. 9 (1), 1911. 10.1038/s41598-019-38500-2 30760781 PMC6374525

[B26] Mauthe M. Orhon I. Rocchi C. Zhou X. Luhr M. Hijlkema K.-J. (2018). Chloroquine Inhibits Autophagic Flux by Decreasing Autophagosome-Lysosome Fusion. Autophagy 14 (8), 1435–1455. 10.1080/15548627.2018.1474314 29940786 PMC6103682

[B27] Ntsapi C. Lumkwana D. Swart C. du Toit A. Loos B. (2018). New Insights into Autophagy Dysfunction Related to Amyloid Beta Toxicity and Neuropathology in Alzheimer's Disease. Int. Rev. Cel Mol Biol 336, 321–361. 10.1016/bs.ircmb.2017.07.002 29413893

[B28] Palmqvist S. Insel P. S. Stomrud E. Janelidze S. Zetterberg H. Brix B. (2019). Cerebrospinal Fluid and Plasma Biomarker Trajectories with Increasing Amyloid Deposition in Alzheimer's Disease. EMBO Mol. Med. 11 (12). 10.15252/emmm.201911170 PMC6895602 31709776

[B29] Potashkin J. A. Bottero V. Santiago J. A. Quinn J. P. (2019). Computational Identification of Key Genes that May Regulate Gene Expression Reprogramming in Alzheimer's Patients. PLoS One 14 (9), e0222921. 10.1371/journal.pone.0222921 31545826 PMC6756555

[B30] Saki G. Eidi A. Mortazavi P. Panahi N. Vahdati A. (2020). Effect of β-asarone in normal and β-amyloid-induced Alzheimeric Rats. aoms 16 (3), 699–706. 10.5114/aoms.2020.94659 PMC7212238 32399120

[B31] Sasaguri H. Nilsson P. Hashimoto S. Nagata K. Saito T. De Strooper B. (2017). APP Mouse Models for Alzheimer's Disease Preclinical Studies. Embo J. 36 (17), 2473–2487. 10.15252/embj.201797397 28768718 PMC5579350

[B32] Satir T. M. Agholme L. Karlsson A. Karlsson M. Karila P. Illes S. (2020). Partial Reduction of Amyloid β Production by β-secretase Inhibitors Does Not Decrease Synaptic Transmission. Alz Res. Ther. 12 (1), 63. 10.1186/s13195-020-00635-0 PMC7251689 32456694

[B33] Shen Y. He D. He L. Bai Y. Wang B. Xue Y. (2020). Chronic Psychological Stress, but Not Chronic Pain Stress, Influences Sexual Motivation and Induces Testicular Autophagy in Male Rats. Front. Psychol. 11, 826. 10.3389/fpsyg.2020.00826 32425863 PMC7203493

[B34] Sun A. Wei J. Childress C. Shaw J. H. Peng K. Shao G. (2017). The E3 Ubiquitin Ligase NEDD4 Is an LC3-Interactive Protein and Regulates Autophagy. Autophagy 13 (3), 522–537. 10.1080/15548627.2016.1268301 28085563 PMC5361608

[B35] Sun J. Feng D. Xi H. Luo J. Zhou Z. Liu Q. (2020). CD24 Blunts the Sensitivity of Retinoblastoma to Vincristine by Modulating Autophagy. Mol. Oncol. 14 (8), 1740–1759. 10.1002/1878-0261.12708 32394616 PMC7400807

[B36] Tanji K. Miki Y. Ozaki T. Maruyama A. Yoshida H. Mimura J. (2014). Phosphorylation of Serine 349 of P62 in Alzheimer's Disease Brain. Acta Neuropathol. Commun. 2, 50. 10.1186/2051-5960-2-50 24886973 PMC4035093

[B37] Torossian A. Broin N. Frentzel J. Daugrois C. Gandarillas S. Saati T. A. (2019). Blockade of Crizotinib-Induced BCL2 Elevation in ALK-Positive Anaplastic Large Cell Lymphoma Triggers Autophagy Associated with Cell Death. Haematologica 104 (7), 1428–1439. 10.3324/haematol.2017.181966 30679328 PMC6601090

[B38] Vélez J. I. Lopera F. Creagh P. K. Piñeros L. B. Das D. Cervantes-Henríquez M. L. (2019). Targeting Neuroplasticity, Cardiovascular, and Cognitive-Associated Genomic Variants in Familial Alzheimer's Disease. Mol. Neurobiol. 56 (5), 3235–3243. 10.1007/s12035-018-1298-z 30112632 PMC6476862

[B39] Wang G. Li Y. Liu J. Yuan Y. Shen Z. Mei X. (2017). Ultrasensitive Multiplexed Immunoassay of Autophagic Biomarkers Based on Au/rGO and Au Nanocages Amplifying Electrochemcial Signal. Sci. Rep. 7 (1), 2442. 10.1038/s41598-017-02766-1 28550286 PMC5446417

[B40] Wang L. Chen M. Yang J. Zhang Z. (2013). LC3 Fluorescent Puncta in Autophagosomes or in Protein Aggregates Can Be Distinguished by FRAP Analysis in Living Cells. Autophagy 9 (5), 756–769. 10.4161/auto.23814 23482084 PMC3669184

[B41] Wang N. Wang H. Li L. Li Y. Zhang R. (2019). β-Asarone Inhibits Amyloid-β by Promoting Autophagy in a Cell Model of Alzheimer's Disease. Front. Pharmacol. 10, 1529. 10.3389/fphar.2019.01529 32009952 PMC6979317

[B42] Wong L. R. Wong P. Ho P. C.-L. (2020). Metabolic Profiling of Female Tg2576 Mouse Brains Provides Novel Evidence Supporting Intranasal Low-Dose Pioglitazone for Long-Term Treatment at an Early Stage of Alzheimer's Disease. Biomedicines 8 (12), 589. 10.3390/biomedicines8120589 PMC7764407 33317213

[B43] Zhang Y. Huang N. Lu H. Huang J. Jin H. Shi J. (2020). Icariin Protects against Sodium Azide-Induced Neurotoxicity by Activating the PI3K/Akt/GSK-3β Signaling Pathway. PeerJ 8, e8955. 10.7717/peerj.8955 32341897 PMC7179568

[B44] Zhang Y. Zhang Y. Jin X.-f. Zhou X.-h. Dong X.-h. Yu W.-t. (2019). The Role of Astragaloside IV against Cerebral Ischemia/Reperfusion Injury: Suppression of Apoptosis via Promotion of P62-LC3-Autophagy. Molecules 24 (9), 1838. 10.3390/molecules24091838 PMC6539971 31086091

[B45] Zhang Z. Zhou L. Xie N. Nice E. C. Zhang T. Cui Y. (2020). Overcoming Cancer Therapeutic Bottleneck by Drug Repurposing. Sig Transduct Target. Ther. 5 (1), 113. 10.1038/s41392-020-00213-8 PMC7331117 32616710

